# Self-Reported Risks for Multiple-Drug Resistance among New Tuberculosis Cases: Implications for Drug Susceptibility Screening and Treatment

**DOI:** 10.1371/journal.pone.0025861

**Published:** 2011-10-14

**Authors:** Timothy F. Brewer, Howard W. Choi, Carlos Seas, Fiorella Krapp, Carlos Zamudio, Lena Shah, Antonio Ciampi, S. Jody Heymann, Eduardo Gotuzzo

**Affiliations:** 1 Department of Medicine, McGill University, Montreal, Quebec, Canada; 2 Department of Epidemiology, Biostatistics & Occupational Health, McGill University, Montreal, Quebec, Canada; 3 Institute of Health and Social Policy, McGill University, Montreal, Quebec, Canada; 4 Department of International Health, Bloomberg School of Public Health, Johns Hopkins University, Baltimore, Maryland, United States of America; 5 Instituto de Medicina Tropical Alexander von Humboldt, Universidad Peruana Cayetano Heredia, Lima, Peru; Fundació Institut Germans Trias i Pujol; Universitat Autònoma de Barcelona CibeRES, Spain

## Abstract

**Background:**

Multiple drug-resistance in new tuberculosis (TB) cases accounts for the majority of all multiple drug-resistant TB (MDR-TB) worldwide. Effective control requires determining which new TB patients should be tested for MDR disease, yet the effectiveness of global screening recommendations of high-risk groups is unknown.

**Methods:**

Sixty MDR-TB cases with no history of previous TB treatment, 80 drug-sensitive TB and 80 community-based controls were recruited in Lima, Peru between August and December, 2008 to investigate whether recommended screening practices identify individuals presenting with MDR-TB. Odd ratios (OR) and 95% confidence intervals (CI) were calculated using logistic regression to study the association of potential risk factors with case/control variables.

**Results:**

MDR-TB cases did not differ from drug-sensitive TB and community controls in rates of human immunodeficiency virus infection, reported hospital or prison visits in the 3 years prior to diagnosis. MDR-TB cases were more likely than drug-sensitive TB controls to have had a recent MDR-TB household contact (OR 4.66, (95% CI 1.56–13.87)); however, only 15 cases (28.3%) reported this exposure. In multivariate modeling, recent TB household contact, but not contact with an MDR-TB case, remained predictive of MDR-TB, OR 7.47, (95% CI 1.91–29.3). Living with a partner rather than parents was associated with a lower risk of MDR-TB, OR 0.15, (95% CI 0.04–0.51).

**Conclusion:**

Targeted drug susceptibility testing (DST) linked to reported MDR-TB contact or other high-risk exposures does not identify the majority of new TB cases with MDR disease in Lima where it is endemic. All new TB cases should be screened with DST to identify MDR patients. These findings are likely applicable to other regions with endemic MDR-TB.

## Introduction

Multiple drug-resistant tuberculosis (MDR-TB) is growing worldwide, and threatens to undermine global tuberculosis (TB) control efforts. [1] In 2008 there were an estimated 440,000 incident MDR-TB cases globally; among those tested, 5.4% of MDR-TB patients had extensively drug-resistant (XDR)-TB. [2], [3] Inappropriate or inadequate TB treatment programs are assumed to be responsible for generating MDR disease, and TB patients with a history of previous treatment are as much as 10 times more likely to have MDR disease than those without previous TB therapy. [4], [5] Despite the well-documented association between previous TB treatment and MDR disease, transmission of MDR-TB, instead of inadequate or inappropriate therapy, is responsible for the majority of cases worldwide. [6]

Even excellent national TB control programs (NTP) based on the Directly-Observed Therapy, Short-course (DOTS) strategy may experience substantial increases in MDR-TB, [6] and observational and modeling studies suggest that DOTS treatment expansion may preferentially select for drug-resistant TB transmission in endemic locations. [7], [8], [9] TB incidence has declined substantially in Peru since the introduction of its NTP in 1990; however, MDR-TB cases grew 27-fold from 1997 to 2005. [10] Understanding MDR-TB transmission is crucial to global TB control efforts, yet little is known about MDR-TB risk factors in new TB cases with either no history or <1 month history of previous TB therapy. [11], [12]

Selected groups such as social, nosocomial or household contacts of MDR-TB patients have been shown to be at high-risk for acquiring MDR disease, [13], [14], [15], [16] and global TB control plans call for targeted screening of selected new TB patients for MDR-TB prevention. [17] However, the percentage of new TB patients with MDR disease likely to be identified by targeted screening is unknown. If global MDR-TB control efforts are to succeed, it is imperative to understand if recommended screening strategies will identify most individuals at risk for MDR disease in TB-endemic locations. We undertook a case-control study to examine MDR-TB risk factors in new TB patients at a well-run NTP in Peru.

## Methods

### Design

This study was a population based case-control study.

### Recruitment

Cases and controls were recruited between August 1, 2008 and December 12, 2008 in the San Juan de Lurigancho (SJL) district of Lima, one of the poorest and most populated districts in Peru. In 2007 SJL accounted for 3.2% of the Peruvian population (898,443 inhabitants), but reported 7.0% (2,044/29,393) of all TB cases and 14.2% (116/818) of all MDR-TB cases. [18] Cases were identified from an unrelated study of rapid diagnostics for MDR-TB in consecutive new TB patients in SJL. All adult patients ≥18 years old with laboratory proven MDR-TB, no history of previous TB treatment and who were being followed in a SJL NTP clinic during the recruitment period were eligible to be cases. Drug-sensitive pulmonary TB controls were randomly selected in blocks of 30 from a database of all TB patients followed in SJL NTP clinics; medical records were reviewed to determine eligibility, which included ≥18 years old, no history of previous TB treatment, sputum smear-negative at 2 months on NTP standard therapy and no treatment failure or relapse at the time of interview. Because sputum cultures and drug susceptibility testing (DST) are not routinely performed in the Peru NTP, response to standard therapy within 2 months of initiating treatment was used as a surrogate for drug-sensitive TB.

To select community controls, a population weighted sampling method based on the 34 NTP clinic catchment areas was developed. Catchment areas were randomly chosen using a computer-generated scheme, where any given area might be selected once, multiple times or not at all. Each chosen catchment area was divided into subdivisions of approximately equal size, and a computer-based random number generator was used to create a list of subdivisions in SJL where the research nurse went to find community controls. Upon arriving at the selected locations, the first adult ≥18 year old encountered and who on questioning did not have a history of prior TB was invited to participate in the study. If that person refused or was not eligible, the interviewer approached another person until someone agreed to participate [19]

All study participants provided informed, written consent before being enrolled. The study was reviewed and approved by the Human Subjects Review Committees of Universidad Peruana Cayetano Heredia (UPCH), the McGill University Health Centre Research Institute and the Ministry of Health Dirección de Salud Lima Este.

### Questionnaire design

Questionnaires were developed to explore reported demographic, socioeconomic and exposure factors associated with MDR-TB. Primary hypotheses included investigating the role of reported healthcare and prison visits before MDR-TB diagnosis and time spent living in SJL where MDR-TB rates exceed much of Lima and Peru. Risk factors associated with education, employment, income, health and personal behaviors, housing conditions, neighborhood characteristics, and transportation also were studied.

Questionnaires were developed in English, translated into Spanish by native Spanish speakers fluent in both languages and then were back-translated by bilingual individuals not involved in the original translations. Discrepancies were resolved by consensus. The questionnaires were pilot tested in 10 UPCH employees for comprehensibility, language level and length of time. After adjustment, the questionnaires were field tested with 10 MDR-TB patients, 10 TB controls and 10 community controls after informed consent. These tests were used to make final adjustments after which no further changes were made. Data from the 40 pilot questionnaires were not used in the analyses. All questionnaires were administered orally in Spanish by trained research nurses or study investigators.

### Statistical Analysis

With 60 MDR-TB cases and a similar number of controls, we anticipated having 80% power to detect odd ratios (OR) of 3.0 or more for risk factors prevalent in at least 15% of the population at a significance level of 0.05. [20] These OR are in the range of commonly reported TB risk factors. [5], [11], [21]

After simple descriptive analysis, the association of potential risk factors with the case/control variables was studied using logistic regression. We computed the crude and adjusted OR with 95% confidence intervals (CI). We used simple univariate logistic regression for categorical and continuous variables to assess the individual correlates of community controls, drug-sensitive TB controls and MDR-TB cases. Multivariate analysis was conducted to fit a model for the dichotomous outcome comparing MDR-TB cases with drug-sensitive TB controls. The adjustment was performed at first by developing a ‘full’ logistic regression model, which included all potential risk factors with crude OR significant at the 10% level. In order to deal with multicollinearity, backward-stepwise variable selection was performed to eliminate redundant covariates. The final model reported was the one minimizing the Bayesian Information Criterion (BIC). [22] The Hosmer-Lemeshow test was used to assess the goodness-of-fit of our final model (minimum BIC). All calculations were performed using R software.

### Role of the funding source

The study sponsors had no role in the study design, implementation, data collection, analyses, interpretation or the writing of this report. The authors had full access to all data and final responsibility for the decision to submit this study for publication.

## Results

Sixty-five newly diagnosed TB patients with MDR disease were followed in SJL NTP clinics during the study period; 60 (92.3%) agreed to participate and were compared with 80 randomly selected drug-sensitive TB controls and 80 community controls. Eighty-two drug-sensitive pulmonary TB patients were approached to enroll 80 controls (97.6% participation rate). Among community controls, 154 individuals were approached in order to enroll 80 controls. Refusals—74 (48.1%)—had less education than participants (p = 0.0003); otherwise there were no significant differences between community participants and non-participants in terms of age, gender or civil status.

There was no significant difference between cases and controls in the number of reported health care center visits or hospitalizations in the 3 years prior to TB diagnosis or interview in the case of community controls, type of health care center visited, length of time spent in waiting rooms, alcohol use, a history of incarceration or prison visits, or length of time living in SJL (Tables 1 and 2). Cases and controls did not report differences in known exposures to neighbors with TB symptoms, TB disease or who had died from TB; however, MDR-TB cases were more likely to report knowing a neighbor who also had MDR-TB than drug-sensitive TB or community controls (13.7% vs. 4.0% vs. 2.5%, p for trend = 0.0016). Eighteen percent of MDR-TB cases reported a previous TB death in the household, compared with 7.6% of TB controls and 1.3% of community controls (p for trend = 0.0022).

**Table 1:** Descriptive statistics of multiple drug-resistant tuberculosis (MDR-TB) cases, drug sensitive tuberculosis (DS-TB) controls and community controls from Lima, Peru, August-December 2008.

	Community Controls Number (%) (n = 80)	DS-TB Controls Number (%) (n = 80)	MDR-TB Cases Number (%) (n = 60)	MDR compared with pooled controls, OddsRatio (95% Confidence Intervals)	p values
**Age in years, mean [s.d.]**	38.6 [13.5]	33.6 [15.3]	29.9 [12.1]	0.97 (0.94–0.99)	0.0070
**Sex**					
Male	42 (52.5)	45 (56.3)	30 (50.0)	Ref	
Female	38 (47.5)	35 (43.8)	30 (50.0)	1.19 (0.66–2.16)	0.5625
**Civil status**					
Co-habiting/Married	50 (62.5)	41 (51.3)	17 (28.3)	Ref	
Single	25 (31.3)	29 (36.3)	38 (63.3)	3.77 (1.94–7.32)	<0.0001
Separated/Widowed	5 (6.3)	10 (12.5)	5 (8.3)	1.78 (0.57–5.56)	0.3182
**Ethnic group**					
Mestizo	72 (90.0)	63 (78.8)	52 (86.7)	Ref	
White	2 (2.5)	6 (7.5)	5 (8.3)	1.62 (0.51–5.19)	0.4144
Quechua	5 (6.2)	8 (10.0)	1 (1.7)	0.20 (0.03–1.57)	0.1252
Other	1 (1.2)	3 (3.7)	2 (3.3)	1.30 (0.23–7.31)	0.7672
**Highest level of education**					
No schooling or Incomplete Primary	4 (5.0)	6 (7.5)	4 (6.7)	Ref	
Complete Primary or Incomplete/Complete Secondary	57 (71.3)	59 (73.8)	36 (60.0)	0.78 (0.23–2.62)	0.6831
Technical or University	19 (23.8)	15 (28.7)	20 (33.3)	1.47 (0.41–5.31)	0.5560
**Vaccinated with BCG** *					
No	2 (2.5)	10 (12.5)	9 (15.3)	Ref	
Yes	74 (92.5)	63 (78.8)	47 (79.7)	0.46 (0.18–1.15)	0.0977
Unsure	4 (5.0)	7 (8.8)	3 (5.1)	0.36 (0.078–1.70)	0.1984
**Housing**					
Rent	17 (21.3)	15 (18.8)	8 (13.3)	Ref	
Own	26 (32.5)	30 (37.5)	23 (38.3)	1.64 (0.66–4.10)	0.2876
Lives with family or friends	37 (46.2)	35 (43.7)	29 (48.3)	1.61 (0.66–3.91)	0.2921
**Length of time living in the district**					
<5 years	9 (11.2)	20 (25.0)	8 (13.3)	Ref	
6–10 years	8 (11.3)	9 (11.3)	10 (16.7)	2.13 (0.71-6.44)	0.1795
11–15 years	8 (10.0)	9 (11.3)	6 (10.0)	1.28 (0.38–4.32)	0.6914
>15 years	55 (68.7)	42 (52.5)	36 (60.0)	1.35 (0.56–3.22)	0.5047
**Number of household members at the time of diagnosis**					
0–2	15 (18.7)	22 (27.5)	10 (16.7)	Ref	
3–6	46 (57.5)	36 (45.0)	30 (50.0)	1.35 (0.60–3.06)	0.4663
>7	19 (23.7)	22 (27.5)	20 (33.3)	1.81 (0.75–4.35)	0.1884
**Unemployed in the 12 months prior to TB Diagnosis**	47 (58.8)	38 (47.5)	30 (50.0)	0.88 (0.49–1.60)	0.6795
**Monthly Household Income (soles)** *					
>600	49 (66.2)	36 (50.7)	26 (49.1)	Ref	
300–600	25 (33.8)	20 (28.2)	20 (37.7)	1.45 (0.73–2.89)	0.2856
<300	0 (0)	15 (21.1)	7 (13.2)	1.53 (0.56–4.14)	0.4072
**Attended hospital in 3 years prior to TB diagnosis**	63 (78.7)	63 (78.7)	43 (71.7)	0.68 (0.31–1.48)	0.3334
**HIV positive**	0 (0.0)	3 (3.7)	0 (0.0)	N/A	N/A
**Immunocompromised** §	9 (11.3)	14 (17.5)	13 (21.6)	1.65 (0.77–3.51)	0.1957
**Depression**	1 (1.3)	1 (1.3)	8 (13.3)	12.15 (2.50–59.04)	0.0020
**Diabetes**	0 (0.0)	3 (3.7)	4 (6.7)	3.74 (0.81–17.22)	0.0907
**Lung disease**	4 (5.0)	3 (3.8)	3 (5.0)	1.15 (0.29–4.60)	0.8430
**Smoking status**					
Never	29 (36.3)	29 (36.3)	30 (50.0)	Ref	
Ex-smoker	19 (23.8)	49 (61.3)	29 (48.3)	0.83 (0.44–1.53)	0.5412
Current	32 (40.0)	2 (2.5)	1 (1.7)	0.057 (0.007–0.44)	0.0058
**Drug use (illicit)**	1(1.2)	12(15.0)	5(8.3)	1.028 (0.35–3.018)	0.9597

*Excludes missing values; N/A – not applicable;

§ On steroids or diagnosed with another medical condition associated with immunodeficiency.

**Table 2:** Comparison of exposure risk factors in multiple drug-resistant tuberculosis (MDR-TB) cases with drug-sensitive tuberculosis (DS-TB) controls from Lima, Peru, August-December, 2008.

	DS-TB Controls, n (%) (n = 80)	MDR-TB Cases, n (%) (n = 60)	Odds Ratio (95% Confidence Limit)	p-values
**In jail in 3 years prior to diagnosis**	7 (8.8)	1 (1.7)	0.18 (0.021–1.48)	0.0740
**Living at the time of diagnosis with:**				
** Mother**	26 (32.5)	33 (55.0)	2.54 (1.27–5.066)	0.0076
** Father**	17 (21.3)	27 (45.0)	3.032 (1.45–6.35)	0.0027
** Grandparent**	4 (5.0)	2 (3.3)	0.66 (0.12–3.70)	0.7003
** Sibling**	28 (35.0)	39 (65.0)	3.45 (1.71–6.96)	0.0004
** Partner**	39 (48.8)	15 (25.0)	0.35 (0.17–0.73)	0.0043
**Shares a bedroom**	55 (68.7)	42 (70.0)	1.061 (0.51–2.20)	0.8739
**No. bedrooms in the house** *				
1–2	34 (43.0)	27 (45.0)	Ref	
3–4	30 (38.0)	19 (31.7)	0.80 (0.37–1.71)	0.5623
>5	15 (19.0)	14 (23.3)	1.18 (0.48–2.85)	0.7210
**Household member in the 3 years prior to TB diagnosis with:**				
** TB Symptoms**	21 (26.2)	25 (41.7)	2.009 (0.98–4.10)	0.0546
** TB Diagnosis**	13 (16.3)	23 (38.3)	3.20 (1.45–7.057)	0.0031
**Anyone else who ever lived in the same house with:** **				
** TB Treatment**				
No	51 (69.9)	35 (58.3)	Ref	
Yes	22 (30.1)	25 (41.7)	1.66 (0.81–3.31)	0.1677
** MDR-TB Diagnosis**				
No	59 (92.2)	38 (71.7)	Ref	
Yes	5 (7.8)	15 (28.3)	4.66 (1.56–13.87)	0.0057
** Death from TB**				
No	70 (92.1)	48 (81.4)	Ref	
Yes	6 (7.9)	11 (18.6)	2.67 (0.93–7.72)	0.0691
**Anyone in the neighborhood with:** **				
** TB Symptoms**				
No	38 (63.3)	26 (55.3)	Ref	
Yes	22 (36.7)	21 (44.7)	1.40 (0.64–3.039)	0.4020
** TB Diagnosis**				
No	39 (63.9)	25 (52.1)	Ref	
Yes	22 (36.1)	23 (47.9)	1.63 (0.76–3.52)	0.2135
** MDR-TB Diagnosis**				
No	38 (92.7)	29 (80.6)	Ref	
Yes	3 (7.3)	7 (19.4)	3.057 (0.73–12.86)	0.1272
** Death from TB**				
No	64 (97.0)	50 (92.6)	Ref	
Yes	2 (3.0)	4 (7.4)	2.56 (0.45–14.55)	0.2889
**Anyone in the Workplace with:** **				
** TB Symptoms**				
No	41 (68.3)	27 (58.7)	Ref	
Yes	19 (31.7)	19 (41.3)	1.51 (0.68–3.38)	0.3062
** TB Diagnosis**				
No	45 (81.8)	25 (75.8)	Ref	
Yes	10 (22.2)	8 (24.2)	1.44 (0.50–4.12)	0.4963
** MDR-TB Diagnosis**				
No	45 (97.8)	23 (88.5)	Ref	
Yes	1 (2.2)	3 (11.5)	5.87 (0.58–59.55)	0.1347
** Death from TB**				
No	61 (98.4)	43 (95.5)	Ref	
Yes	1 (1.6)	2 (4.4)	2.84 (0.25–32.29)	0.4007
**Workplace environment** *				
Exterior	28 (45.1)	15 (27.2)	Ref	
Interior	34 (54.8)	40 (72.2)	2.20 (1.011–4.72)	0.0470
**Number of people working in the same room** *				
0	28 (45.2)	17 (30.9)	Ref	
1–4	16 (25.8)	14 (25.4)	1.44 (0.57–3.68)	0.4445
>5	18 (29.0)	24 (43.6)	2.20 (0.93–5.18)	0.0724

*Excludes missing values;

**Excludes unsure responses.

There were no significant differences among MDR-TB cases and drug-sensitive TB or community controls in reported ethnicity, level of education, household income, illicit drug use or reported history of Bacille Calmette-Guérin vaccination. Only 3 TB controls (3.7%) were human immunodeficiency virus (HIV) co-infected. Cases and controls did not differ in the percentage with heart, lung or renal disease, other causes of immunosuppression or diabetes. MDR-TB cases were younger than drug-sensitive TB or community controls (29.9 vs. 33.6 vs. 38.6 years, p = 0.0011), and were more likely to be single (Table 1).

In bivariate analyses drug-sensitive TB controls were more likely than MDR-TB cases to live at home, to report a household contact diagnosed with TB in the last 3 years or to report a household contact with MDR-TB, yet only 15 cases (28.3%) reported household contact with a previous MDR-TB patient. Cases also were more likely to have worked indoors than drug-sensitive TB controls. Having a household contact with TB symptoms was borderline significant for MDR disease (Table 2).

In multivariate modeling comparing MDR-TB cases with drug-sensitive TB controls, having a household member with a TB diagnosis in the past three years remained predictive of MDR-TB, odds ratio (OR) 7.47, (95% CI 1.91–29.3). Living with a partner rather than parents was associated with a lower risk of MDR disease, OR 0.15, (95% CI 0.04–0.51) (Table 3). The Hosmer-Lemeshow test statistic to assess the goodness-of-fit of our final model was 3.899 based on 8 degrees of freedom, which corresponds to a p-value of 0.867, indicating a very good fit.

**Table 3:** Multivariate comparison of exposure risk factors in multiple drug-resistant tuberculosis (MDR-TB) cases with drug-sensitive tuberculosis (DS-TB) controls.

	Univariate Analysis Odds ratio (OR) (95% confidence intervals)*	Full Model OR (95% CI)	Best Bayesian Information Criterion Model OR (95% CI)
**Living at the time of diagnosis with:**			
** Mother**	2.29 (0.99–5.27) †	0.07 (0.003–1.85)	
** Father**	2.73 (1.14–6.52) ‡	3.89 (0.54–28.1)	
** Sibling**	4.21 (1.77–10.04) ‡	15.4 (1.15–205) ‡	
** Partner**	0.25 (0.10–0.65) ‡	0.11 (0.01–0.97) ‡	0.15 (0.04–0.51) ‡
**Household member in the 3 years prior to TB diagnosis with:**			
** TB Symptoms**	2.27 (0.93–5.53) †	0.08 (0–12.63)	
** TB Diagnosis**	4.37 (1.60–11.97) ‡	4.1 (0.3–18527)	7.47 (1.91–29.3) ‡
**Anyone else who ever lived in the same house with:** §			
** TB Treatment**	2.27 (0.93–5.53) †	0.024 (0–5.42)	
** MDR**–**TB Diagnosis**	3.27 (0.92–11.55) †	9.96 (0.59–1672)	
** Death from TB**	3.36 (0.81–13.93)	3.68 (0.041–333)	

*Study participants with missing observations for variables retained in multivariate model were excluded.

§ Excludes missing values or unsure responses.

† p≤0.10;

‡ p≤0.05.

## Discussion

Despite the expansion of DOTS control programs worldwide, MDR-TB—particularly MDR disease in persons with no history of previous TB treatment—is increasing. For targeted DST to be an effective strategy for containing the growing MDR-TB epidemic, it is imperative to determine whether self-reported risk factors will identify new TB patients with MDR disease.

We describe one of the largest population-based case-control studies to date of MDR-TB risk factors in new TB patients and include community controls. Peru has a long-standing, comprehensive NTP that provides therapy to essentially all TB patients while achieving WHO detection and treatment targets. [23] The NTP case detection rate was 93% in 2007, and 92% of patients were successfully treated; in contrast to some sub-Saharan African countries, few TB patients are HIV co-infected. [18], [24] Passive case detection using sputum smears is done to screen patients for TB. Sputum cultures are routinely performed only in HIV co-infected individuals, healthcare workers, contacts of MDR-TB patients, retreatment or smear-negative cases. Patients who remain smear-positive after 2 months of NTP standard TB treatment also are referred for sputum culture and DST. Newly presenting TB patients who fail NTP standard therapy here are at high-risk for having culture-positive MDR disease. [25], [26]

Our results demonstrate that MDR-TB in new cases in this setting are not easily distinguished from drug-sensitive TB patients or community controls by self-reported socio-economic or demographic factors or time spent in potentially high-risk settings such as hospitals or prisons. MDR-TB cases were more likely than drug-sensitive TB controls to report having had a recent household contact with TB or MDR-TB; however, only 28.3% of new cases with MDR disease reported exposure to a household contact with MDR-TB. MDR-TB cases were younger than controls, and were more likely to live at home. In multivariate modeling, living with a partner remained protective against having MDR-TB, OR 0.15, (95% CI 0.04–0.51). Detecting MDR disease in this setting is time-consuming, usually requiring at least 3 to 6 months to make a diagnosis. [27] Delays in identifying and treating MDR-TB in Lima could contribute to the observation that these patients were more likely to live with family as their extended illness may affect their ability to work or support themselves independently. However, other possible explanations for the association between MDR-TB disease and living at home, such as younger age, exist. Additional study is needed to understand the reason for this association.

These findings have important implications for TB control in SJL and similar locations where MDR disease exists. First, they suggest that MDR-TB in this population is an endemic disease, not confined to outbreaks or special groups. Second, they suggest that TB control efforts need to be rethought for all patients, not only those presumed to be at high-risk for MDR-TB. Identifying high-risk groups for selected DST is currently practiced in Peru and recommended by global TB control plans. [1], [17] While potentially useful, [28] selected DST is unlikely to control MDR-TB transmission in SJL. Existing recommendations for DST in new TB patients would have missed almost three-quarters of all new cases of MDR-TB in this district. Effective TB control in SJL will likely at a minimum entail introducing routine DST for all new patients with a history of recent TB contact. More likely universal DST in new TB patients will be needed, a recommendation applicable to other areas with similar high rates of MDR disease such as Shanghai, China. [29]

The lack of timely diagnostic tests for MDR-TB leads to substantial delays in the identification and treatment of MDR-TB patients, and may contribute to MDR-TB transmission in communities. [30] A number of diagnostic methods have been developed which show promise in rapidly recognizing MDR-TB, including microscopic-observation drug susceptibility assays (MODS) and automated real-time polymerase-chain-reaction based tests. [31], [32] MODS uses inverted light microscopy to observe characteristic cord formation of *Mycobacterium tuberculosis* growth in liquid-culture media; drug susceptibility is determined by comparing growth in drug-containing wells with growth in drug-free wells. [31] Reported advantages of MODS include the reduced time to *M. tuberculosis* diagnosis including drug susceptibility results and safety relative to other indirect susceptibility testing methods. [33]

Xpert MTB/RIF uses an automated real-time polymerase-chain-reaction to amplify and to detect a segment of the *M. tuberculosis* specific *rpoB* gene; [32] this diagnostic test has been shown to be more accurate than existing protocols for identifying TB patients under field conditions in TB-endemic settings, and in smear-negative patients is capable of significantly reducing the time to appropriate treatment. [34] This test also identifies rifampin resistance, a reasonable surrogate marker for MDR-TB. Being able to more rapidly recognized MDR-TB patients has been associated with substantial declines in time to appropriate therapy for MDR-TB patients, [35] and may reduce transmission. [36] The expanded use of this or similar tests may increase laboratory costs for TB diagnosis. However, these tests may be cost-effective overall if they improve diagnostic accuracy and reduce time to inappropriate treatment. [37]

Study strengths include the use of DST in consecutive new TB patients to identify MDR-TB cases. The high participation rate of cases and TB controls in this study meant that they are likely representative of new MDR-TB and drug-sensitive TB patients in SJL. Cases and controls were drawn from the entire district, and not limited to a single institution or referral center. The inclusion of community controls enabled us to explore risk factors for MDR disease separate from those for TB. Study limitations include the lack of drug-susceptibility data for TB controls and the moderate refusal rate among community controls. The lack of DST among TB controls means that some MDR-TB patients may have been misclassified as having drug-susceptible disease. [26] This misclassification, if it occurred, would have had the effect of reducing differences between the two groups. Except for a higher level of education, community controls were similar to community members who declined to participate in the study. Cases and controls were similar across a range of socioeconomic indicators; it seems unlikely that a lower community control refusal rate would have substantially affected the findings.

Case-control studies are subject to recall bias and confounding. Public health programs based on self-reported risk factors such as selected DST are subject to the same risks of reporting errors and misclassifications. Our results likely reflect what is achievable in existing NTP DST screening programs. Confounding was addressed in the multivariate models, though residual confounding could exist.

This case-control study of self-reported MDR-TB risk factors in the setting of an excellent NTP, low HIV co-infection rates and a substantial burden of MDR disease suggests that the existing TB control of sputum smears for diagnosis, standard treatment for new TB cases and selected DST of high-risk groups identified through self-reporting is insufficient to control MDR-TB once established. DST screening in new TB patients will need to be expanded if MDR-TB control is to be achieved in this setting.
